# Estimating bone mass in children: can bone health index replace dual energy x-ray absorptiometry?

**DOI:** 10.1007/s00247-018-4309-3

**Published:** 2018-11-24

**Authors:** Khalaf Alshamrani, Fabrizio Messina, Nick Bishop, Amaka C. Offiah

**Affiliations:** 1 Department of Oncology and Metabolism, Academic Unit of Child Health, University of Sheffield, Damer Street, Sheffield, S10 2TH UK; 2 Leeds Institute of Clinical Trials Research, University of Leeds, Leeds, UK; 3 Sheffield Children’s NHS Foundation Trust, Western Bank, Sheffield, UK

**Keywords:** Bisphosphonates, Bone health index, Bone mineral density, Children, Dual energy x-ray absorptiometry

## Abstract

**Background:**

Bisphosphonates have been shown to increase metacarpal cortical width. Bone health index is computed from hand radiographs by measuring cortical thickness, width and length of the three middle metacarpals, and may potentially help predict fracture risk in children.

**Objective:**

To compare bone health index with bone mineral density as measured from dual energy X-ray absorptiometry scans in patients with and without bisphosphonate treatment.

**Materials and methods:**

Two hundred ninety-three Caucasian patients (mean age: 11.5±3.7 years) were included. We documented absolute values and z-scores for whole-body less head and lumbar spine bone mineral density then correlated these with the bone health index, which were acquired on the same day, in different patient groups, depending on their ethnicity and diagnosis.

**Results:**

Bone health index showed moderate to strong correlation with absolute values for whole-body (*r*=0.52) and lumbar spine (*r*=0.70) bone mineral density in those not treated with bisphosphonates and moderate correlation absolute values for whole-body (*r*=0.54) and lumber spine (*r*=0.51) bone mineral density for those treated with bisphosphonates. There was weak correlation of z-scores, ranging from *r*=0.11 to *r*=0.35 in both groups.

**Conclusion:**

The lack of a strong correlation between dual energy X-ray absorptiometry and bone health index suggests that they may be assessing different parameters.

## Introduction

Assessment of bone mineral density and bone quality is essential to diagnose patients with diseases affecting the skeleton. In children, the reference standard for assessing bone mineral density is dual energy x-ray absorptiometry. Dual energy x-ray absorptiometry is a valuable tool in patient management, where bone mineral density is assessed at appropriate intervals to monitor response to therapy in patients with low bone mass [1]. Bisphosphonates are commonly used in such patients (e.g., those with osteogenesis imperfecta) and have been shown to increase cortical width [2]. However, dual energy x-ray absorptiometry values are influenced by bone size; therefore, bone mineral density is usually underestimated in children with small bones and overestimated in children with large bones because the depth of the bone is not accounted for [3]. Additionally, dual energy x-ray absorptiometry cannot predict fracture risk in children. Rather, it forms part of a comprehensive skeletal health assessment to monitor patients with low bone mineral density.

During the last three decades, quantitative bone imaging techniques have been improved and tools for analysing images have been developed. One of these methods is radiogrammetry, where the middle phalangeal width and cortical thickness are measured and results are presented as the cortical index [4]. Computer software developed specifically for children automatically calculates bone age and bone mass [5]. The software measures the cortical thickness, width and length of the three middle metacarpals and results are expressed as the bone health index. The software also provides a standard deviation score, which enables comparison with healthy Caucasian children. A small number of studies suggest a potential role for the use of bone health index in assessing bone health in children [6–8]. However, there are limitations to these studies, including small participant numbers [6, 7] and an extended interval of up to 8 months between dual energy x-ray absorptiometry and radiographs [8]. Patients on bisphosphonate therapy were not included in previous studies, yet this group may benefit the most, given that bisphosphonates increase cortical thickness, the very parameter on which the bone health index is based.

The aim of this study is to compare bone health index with bone mineral density dual energy x-ray absorptiometry readings acquired on the same day for different clinical reasons in a large cohort of children, including children on bisphosphonate treatment.

## Materials and methods

All procedures performed in this study were in accordance with the ethical standards of our institution. For this study, formal patient consent and research ethics committee approval were not required.

### Patient selection

We retrospectively identified dual energy x-ray absorptiometry scans and left-hand radiographs of patients who attended Sheffield Children’s NHS Foundation Trust Hospital, United Kingdom, between February 2010 and January 2017. The following inclusion criteria were applied: (1) patients 5–18 years old and (2) dual energy x-ray absorptiometry scans and hand radiographs obtained on the same day.

### Hand radiography and dual energy x-ray absorptiometry

**Fig. 1:**
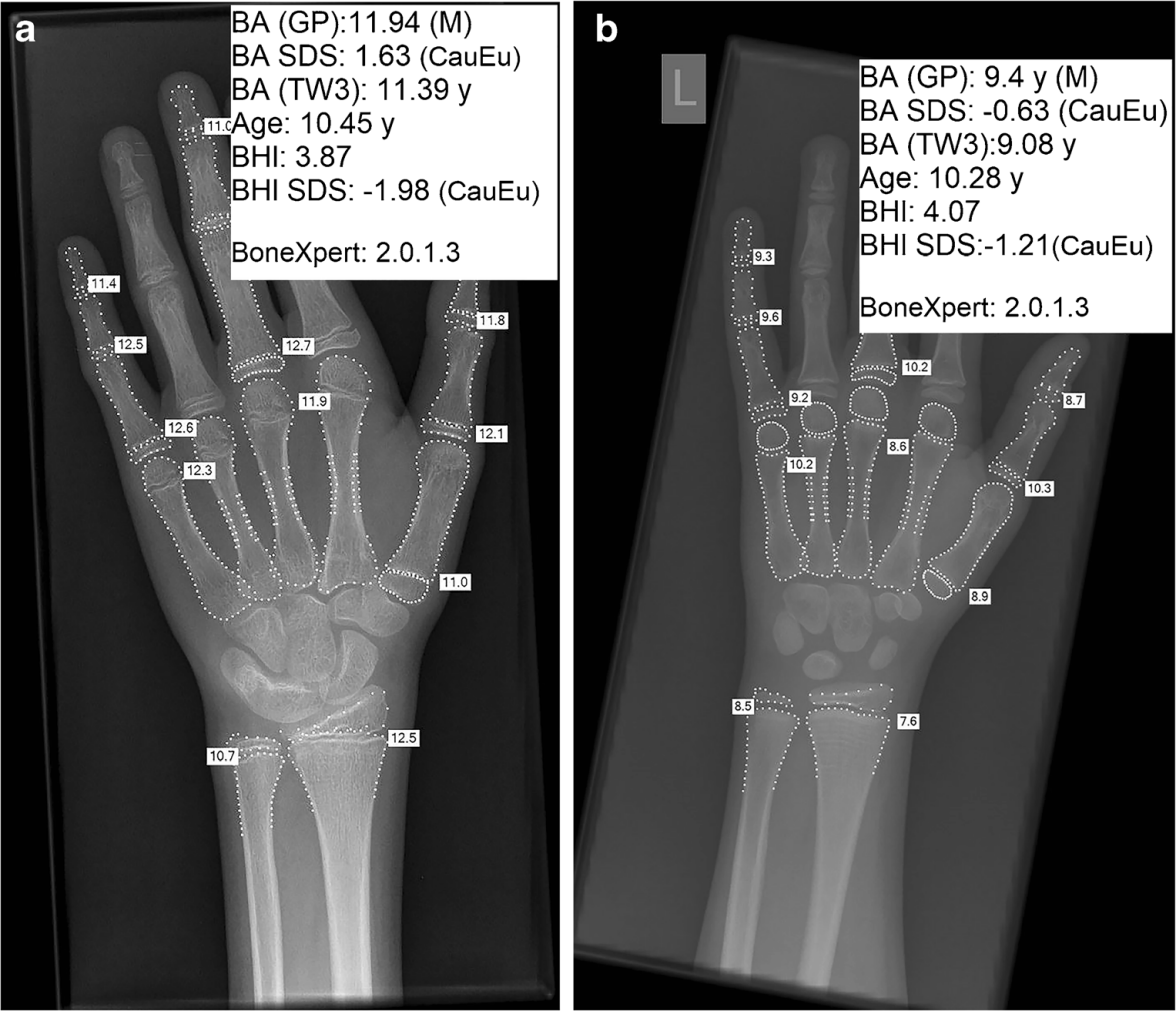
Left-hand radiographs in two 10-year-old Caucasian boys show relatively similar bone health index: (**a**) bisphosphonate naïve and (**b**) on bisphosphonate treatment. *BA (GP)* bone age using Greulich and Pyle’s atlas, *BHI* bone health index, *SDS* standard deviation score, *TW3* Tanner-Whitehouse 3

BoneXpert software (PACS Server version; Visiana, Holte, Denmark) was used to analyse the hand radiographs [5]. All radiographs were in DICOM format. The software calculated the bone health index based on cortical thickness, width and the length of the three middle metacarpals (Fig. 1).

For bone health index calculations, Caucasian was the default ethnicity at the time of analysis. The data was analysed according to whether patients were or were not on bisphosphonate treatment. Cases were excluded from the study if the BoneXpert software was unable to read the radiograph.

Area bone mineral density of total body less head and lumber spine L1-L4 were extracted from each patient’s dual energy x-ray absorptiometry scan. These values were adjusted for age and gender based on normative data provided by the manufacturer. Patient age, gender and the indication for dual energy x-ray absorptiometry were extracted.

### Statistical analysis

Statistical analysis was performed using SPSS version 25 for PC (IBM, Armonk, NY). The z-scores of bone mineral density of the total body less head and spine were adjusted for bone age to evaluate the impact of this adjustment on correlation with the bone health index standard deviation score. Each z-score adjusted for bone age for those patients treated with bisphosphonates is based on the computed z-score values (i.e. the internally standardised residuals from the regression analysis that includes bone age) from the untreated patients. The correlation between bone health index and bone mineral density of the total body less head and the spine was assessed separately using Pearson’s correlation. Additionally, correlation between bone health index standard deviation score and z-score of bone mineral density of the total body less head and the spine was assessed separately. The correlation between the adjusted z-scores and bone health index standard deviation score were then determined. The strength of the correlations was interpreted according to Evans [9], in which the correlation is deemed to be very weak when the r value is less than 0.19, weak between 0.20 and 0.39, moderate between 0.40 and 0.59, strong between 0.60 and 0.79, and very strong between 0.80 and 1.0. Finally, we generated Bland-Altman plots to graphically illustrate the strength of agreement between the two modalities for the non-bisphosphonate and bisphosphonate groups.

## Results

### Patient characteristics

Initially, 577 dual energy x-ray absorptiometry/radiograph pairs were identified. Diagnoses included osteogenesis imperfecta (51%), primary osteoporosis (9.5%) and recurrent fracture (5.8%). All diagnoses/indications and patient characteristics are presented in Tables 1 and 2, respectively.

**Table 1:** Diagnosis or indication for investigation

	No bisphosphonate treatment	Current/past bisphosphonate treatment
Acute back pain	4	
Bone marrow transplant	7	
Calcinosis cutis	6	
Cerebral palsy	9	
Crohn disease	5	3
Cystic fibrosis	9	4
Fanconi anemia	3	
Growth delay	13	
Hypocalcemia	6	
Hypophosphatasia	4	
Juvenile arthritis	10	8
Malabsorption	4	
Osteogenesis imperfecta	12	138
Post colectomy	3	
Primary osteoporosis	15	13
Recurrent fracture	11	6
Total	121	172

**Table 2:** Dual energy x-ray absorptiometry and bone health index measurements

	Bisphosphonate group mean (standard deviation)	Non-bisphosphonate group mean (standard deviation)
Number	172	121
Age (years)	12 (3.5)	11.0 (4.0)
Bone age* (years)	12 (3.7)	9.9 (4.3)
Bone mineral density-spine	0.82 (0.18)	0.83 (0.23)
Z-score of bone mineral density-spine	−0.77 (1.5)	−0.26 (1.6)
Adjusted z-score of bone mineral density-spine	0.0 (1.0)	0.5 (1.3)
Bone mineral density-total body	0.86 (0.16)	0.77 (0.19)
Z-score of bone mineral density-total body	−0.62 (1.4)	−0.43 (1.4)
Adjusted Z-score of bone mineral density-total body	0.0 (1.0)	−0.42 (1.1)
Bone health index	4.4 (0.61)	4.2 (0.68)
Bone health index standard deviation score	−1.2 (1.2)	−1.3 (1.5)

*Greulich and Pyle method

BoneXpert could not interpret 31 (5.6%) radiographs for a number of reasons, including abnormal bone shape, cortical inconsistencies, inconsistencies in length and the image being too sharp. A total of 189 dual energy x-ray absorptiometry/radiograph pairs were excluded as these pairs were acquired for follow-up, which would bias statistical analyses. No dual energy x-ray absorptiometry/radiograph pair was identified for Africans in comparison to a total of 32 dual energy x-ray absorptiometry/radiograph pairs for Asians. However, the Asian patients were excluded from the analysis due to the small number of dual energy x-ray absorptiometry/radiograph pairs identified. Therefore, the final analysis included dual energy x-ray absorptiometry and hand radiographs of 293 patients, 172 (59%) of whom had received bisphosphonate treatment.

### Dual energy X-ray absorptiometry and bone health index

As an overall analysis, bone health index correlated moderately with the absolute values of bone mineral density, the total body and the spine (*P*<0.01) (Table 3). The data were then divided into two groups depending on whether patients had received bisphosphonate treatment. As seen in Table 3, correlation was stronger in the non-bisphosphonate group as depicted by bone mineral density of the total body (*r*=0.70) and the spine (*r*=0.52, *P*<0,01).

**Table 3:** Correlation coefficients between bone health index and dual energy x-ray absorptiometry, and bone health index standard deviation scores and z-score reads in bisphosphonate naive and treated patients

		Overall	*P*-value	Bisphosphonate group	*P*-value	Non-bisphosphonate group	*P*-value
Bone health index	Bone mineral density-spine	0.59	<0.01	0.52	<0.01	0.70	<0.01
	Bone mineral density-total body	0.53	<0.01	0.54	<0.01	0.52	<0.01
Bone health index standard deviation scores	Z-score of bone mineral density-spine	0.17	<0.01	0.047	0.26	0.35	<0.01
	Z-score of bone mineral density-total body	0.24	<0.01	0.19	0.20	0.31	<0.01
Bone health index standard deviation scores	Z-score of bone mineral density-spine (adjusted for bone age)	0.22	0.03	0.12	0.12	0.35	<0.01
	Z-score of bone mineral density-total body (adjusted for bone age)	0.26	<0.01	0.26	<0.01	0.25	<0.01

**Fig. 2:**
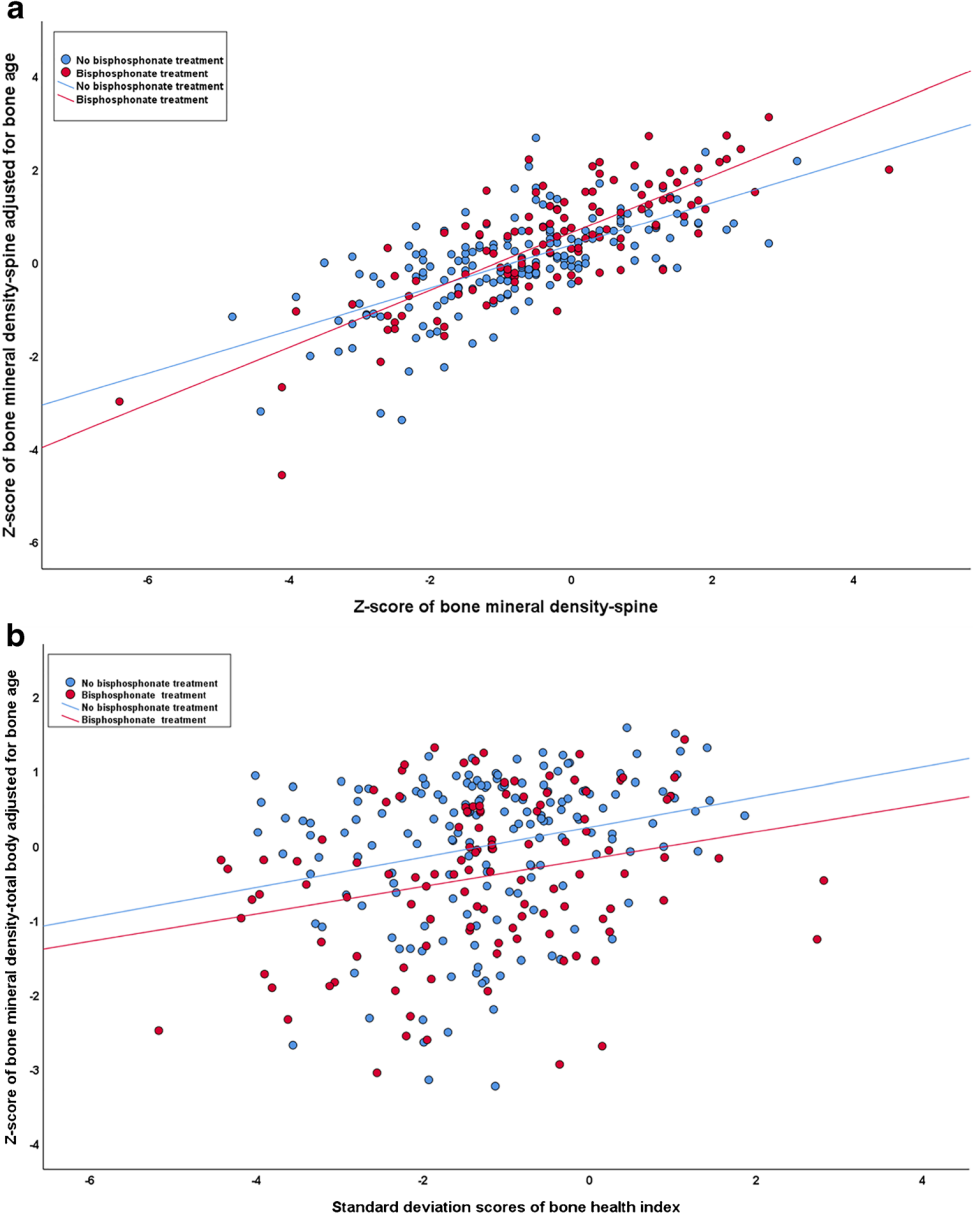
The relationship of z-score adjusted for age and gender alone, and z-score adjusted for age, gender and bone age show similar slopes: (**a**) bone mineral density of the spine and (**b**) bone mineral density of the whole body

**Fig. 3:**
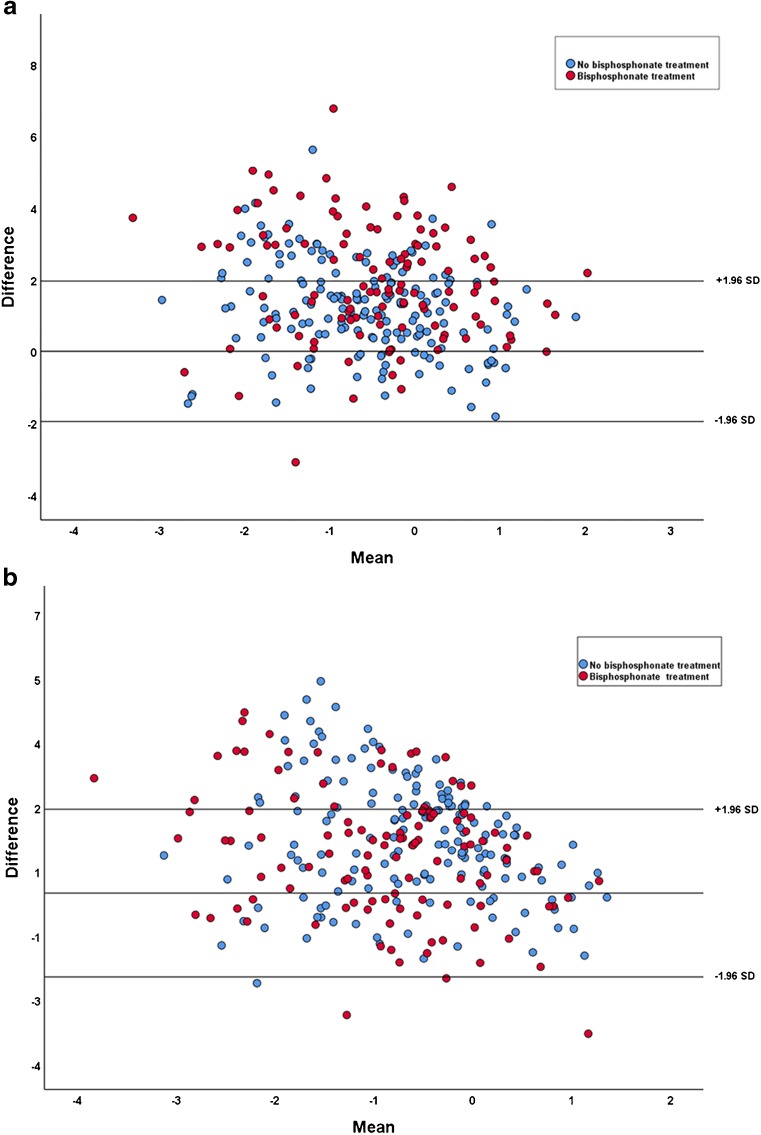
Bland-Altman plots for the difference in bone mineral density adjusted for bone age, and bone health index z-score, versus the mean of the two estimates. **a** Bone mineral density of the spine. **b** Bone mineral density of the whole body. The plots show limited agreement between z-scores of bone mineral density of the total body less head and of the spine

The bone health index standard deviation score showed weak correlation with z-score of the total body less head and the spine (adjusted only for age and gender) in both groups (Table 3). The z-score of bone mineral density of the total body less head and the spine were then adjusted for bone age. The relationship of bone mineral density of the spine adjusted for age and gender alone and adjusted for age, gender and bone age showed similar slopes in both groups with Pearson correlation of 0.74 (*r*^*2*^=0.54) (Fig. 2). Additionally, the relationship of bone mineral density of the total body less head adjusted for age and gender alone and adjusted for age, gender and bone age showed similar slopes in both groups, with Pearson correlation of 0.459 (*r*^*2*^=0.21%) (Fig. 2). The bone health index standard deviation score showed weak correlation with the z-score of bone mineral density of the total body less head and the spine (adjusted for age, gender and bone age) (Table 3). Bland-Altman plots showed limited agreement between z-score of bone mineral density of the total body less head and the spine (adjusted for age, gender and bone age) and bone health index standard deviation scores (Fig. 3).

## Discussion

This study compares bone mineral density measured by dual energy x-ray absorptiometry with bone mass calculated by BoneXpert in a cohort of Caucasian children. BoneXpert was able to provide a reading in the majority of cases. For bisphosphonate naïve children, there was strong correlation between bone health index and dual energy x-ray absorptiometry absolute values. Previous studies have shown similar correlation ranging from *r*=0.58 to *r*=0.85, although ethnicity of patients was not mentioned [6–8].

BoneXpert also provides a bone health index standard deviation score based on data collected from healthy Caucasian children. The bone health index standard deviation score provides a measure of the extent to which a patient’s bone mass is deviated from that of healthy Caucasian children of the same bone age and gender. We found a weak correlation between the bone health index standard deviation score and z-scores of dual energy x-ray absorptiometry, even after adjusting the z-scores for bone age. The reasons for this are uncertain but might include differences in other parameters of reference and study populations. Bland-Altman plots showed systematic bias in which differences are higher than means when means are lower, and the differences do not reach zero until the average value reaches or exceeds two standard deviations. However, this is more likely to be due to the fact that the data adjusted for bone age are based on the computed z-score values from patients who had no bisphosphonate treatment.

The bone health index of patients who had not been on bisphosphonate treatment showed a strong correlation, which might suggest that bone health index is a useful tool to monitor children’s bone health in this group of patients. In the bisphosphonate group, bone health index showed moderate correlation with absolute dual energy x-ray absorptiometry measures. Approximately 79% of the bisphosphonate group were patients with osteogenesis imperfecta. The metacarpals of that group of patients have smaller bone thickness (external size) and thinner cortices than normal [10]. During treatment with bisphosphonates, cortical thickness increases [11]. This is likely to offer BoneXpert an advantage in this particular group of patients, as the bone health index measured by BoneXpert is dependent on cortical structure, while dual energy x-ray absorptiometry depends on both cortical and trabecular structures. The weaker correlation between bone health index and dual energy x-ray absorptiometry in this group of patients may be because bone health index more closely reflects the true state of the children’s bones than dual energy x-ray absorptiometry and merits studies to assess its role in predicting fracture risk in children.

There are some limitations to this study. Firstly, dual energy x-ray absorptiometry z-scores were adjusted for gender, age and ethnicity but not for height and weight. Adjusting for height and weight is expected to explain some of the variance because dual energy x-ray absorptiometry reads may be affected by those parameters. Additionally, dual energy x-ray absorptiometry z-scores adjusted for bone age are based on the computed z-score values from patients who had no bisphosphonate treatment. Ideally, these scores should be based on the z-scores of a healthy population, which were not available to the authors.

## Conclusion

The lack of a strong correlation between dual energy x-ray absorptiometry and bone health index suggests that they may be assessing different parameters. The role of bone health index in assessing bone health in children warrants further study before it can be used as an adjunct to or replacement for dual energy x-ray absorptiometry. Future studies need to investigate the clinical use of the bone health index values for predicting fracture risk.
